# Drought and armed conflict as drivers of overcrowding in Sudan’s refugee camps

**DOI:** 10.1093/inthealth/ihag002

**Published:** 2026-01-31

**Authors:** Safa Abdalrhim, Gorashey Ahmed, Moayad Mudawi, Abobakr Abdulbagi Omer Mohamed, Omaima Mohamed, Iyas Dawood, Rawa Badri

**Affiliations:** Research and Development, End the Neglect Initiative, Khartoum 1111, Sudan; Faculty of Medicine, National Ribat University, Khartoum 1111, Sudan; Research and Development, End the Neglect Initiative, Khartoum 1111, Sudan; Faculty of Medicine, Omdurman Islamic University, Khartoum 1111, Sudan; Research and Development, End the Neglect Initiative, Khartoum 1111, Sudan; Faculty of Medicine, Elrazi University, Khartoum 1111, Sudan; Research and Development, End the Neglect Initiative, Khartoum 1111, Sudan; Faculty of Medicine, University of Khartoum, Khartoum 1111, Sudan; Research and Development, End the Neglect Initiative, Khartoum 1111, Sudan; Faculty of Medicine, Al Neelain University, Khartoum 1111, Sudan; Research and Development, End the Neglect Initiative, Khartoum 1111, Sudan; Faculty of Medicine, Omdurman Islamic University, Khartoum 1111, Sudan; Research and Development, End the Neglect Initiative, Khartoum 1111, Sudan; Mycetoma Research Centre, University of Khartoum, Khartoum 1111, Sudan

**Keywords:** Climate Health, Drought, Refugee Camps, Sudan, Armed Conflict, Overcrowding

## Abstract

Forced migration is a result of variable changes, including human-made factors, environmental factors or both. Climate change has been a significant cause of increased forced migration in Sudan. The prolonged drought attributed to climate change affects Sudanese citizens, with 80% of the population relying on agriculture and pastoralism as their main source of income. Since April 2023, the conflict has displaced over 14.5 million people. Four million have sought safety in neighbouring countries. The refugee camps lack essential resources, and the displaced people face a health crisis, with outbreaks of cholera, 30% of children facing malnutrition and widespread mental health issues. Access to sustainable WASH (water, sanitation and hygiene) systems, improved healthcare, mass vaccinations and climate-sensitive interventions are key to mitigation efforts. The ongoing conflict in Sudan demands a coordinated response from the Sudanese government, humanitarian agencies and international partners to prevent further catastrophe.

## The effects of drought on forced migration and displacement

While drought arises from a complex mix of environmental, political and social factors, climate change serves as a critical threat multiplier, significantly increasing its frequency and severity across Africa. Land degradation and desertification pose a significant environmental concern for 32 African nations.^1^

Sudan’s position between tropical and desert regions means slight rainfall variations are devastating.^2^ Rainfall in Sudan has transformed from predictable seasonal rains to erratic fluctuations, with North Darfur witnessing 20 of its 25 driest years since 1972. In El Fashir, rainfall has dropped from 300 mm annually to approximately 200 mm. This sharp decline has transformed occasional dry periods into extended droughts, primarily impacting the Northern and Western Darfur.^2^

More than 80% of the Sudanese population depends on agriculture and pastoralism as their primary mode of subsistence.^2^ In recent decades, prolonged drought periods have led to a decline in agricultural productivity, forcing pastoralists and farming communities to move to areas with more favourable conditions.^2^ In addition, declining water availability and quality, escalating disputes over water resources, and the desiccation of certain rivers and small water storage facilities have led to widespread livestock mortality.^1^ These factors have intensified the competition between the pastoralists and farmers, which could escalate into political conflict leading to targeted massive displacement.^2^

The current conflict, which began in 2023, is best understood as a continuation and intensification of earlier civil wars in Darfur, South Sudan, Blue Nile and South Kordofan.^3^ This armed conflict is considered today the leading and dominant driver of displacement in Sudan. According to the International Organization for Migration, around 11.3 million people are now internally displaced, with more than 8.5 million forced from their homes since April 2023. Furthermore, around 3.9 individuals crossed into neighbouring countries in the same period.^3^

Preexisting vulnerabilities in Sudan are significantly amplified by the impacts of drought and conflict in the country; these factors precipitated forced migration and displacement, positioning Sudan as the country with the most displaced people worldwide.^2^

## Sudan’s refugee camps and the overcrowding’s health consequences

Sudan’s ongoing armed conflict since April 2023 has displaced over 14.5 million people, and approximately 4 million have fled to neighbouring countries,^4^ including 22% to Egypt, 31% to South Sudan, 39% to Chad, 7% to Ethiopia, and smaller proportions to Libya and the Central African Republic.^5^ These mass displacements have created severely overcrowded refugee camps and settlements, and resulted in consequences that triggered critical health crises, including widespread communicable diseases (cholera, respiratory disease) and non-communicable diseases, malnutrition among children and mental health conditions.

Overcrowding and unsanitary conditions supported the rapid transmission of diseases prone to epidemics.^6^ In addition, limited access to healthcare impedes an effective response. Overcrowding has been a major issue, increasing vulnerability to communicable diseases like cholera. In 2023–2024, a significant cholera outbreak was reported across 10 states in Sudan. The war has worsened the situation by spreading cholera through the destruction of infrastructure, the displacement of people and the disruption of healthcare, resulting in 9581 confirmed cases and 238 deaths.^6^ In addition, outbreaks of malaria (2 679 314 cases; 134 deaths), measles (903 cases; 13 deaths) and dengue (11 449 cases; 20 deaths) have placed a threat to an already fragile health system.^4^

Malnutrition, especially among children, who are estimated to comprise 50–60% of Internally Displaced Person (IDPs). UNICEF recently reported that the rate of child malnutrition has risen to 30%, due to the ongoing conflict.^5^ In response, WHO Sudan expanded nutrition services in conflict-affected areas, admitting 4252 children with severe acute malnutrition to stabilization centres, with 3914 successfully treated.^4^ Nevertheless, recent evidence suggests nearly 7 in 10 children were affected by malnutrition.^7^

Long-term consequences of overcrowded camps extend beyond immediate disease outbreaks. Mental health issues, including depression (18%), Post-Traumatic Stress Disorder (PTSD) (20%) and anxiety (14%), are prevalent among displaced populations.^7^ These psychological impacts are exacerbated by overcrowded living conditions that provide little privacy or dignity.

## Recommendations and future actions

Sudan’s refugee camps need sustainable interventions to reduce the effects of drought on the refugees. Refugees are experiencing a wide range of problems related to water shortage, overcrowding, climate change, etc. Efforts must be made to reduce the problem in both short-term and long-term ways. One of the priorities is to implement WASH (water, sanitation and hygiene) infrastructure across refugee camps, through the installation of solar-powered boreholes, less costly and environmentally agreeable compared with diesel.

In addition, they contribute to the sustainability of water resources through the smart water sensors, which provide data for water conservation and efficient resource management. Furthermore, hygiene education in camps, proper wastewater management, constructing biogas facilities and expanding access to latrines can help reduce the spread of waterborne diseases, such as cholera.^8^

Additionally, when climate information is combined with displacement patterns, organizations are able to anticipate future needs and tactics in order to plan where to allocate resources so that camps are better equipped to handle the pressures brought on by climate change. This approach will enhance sustainability, improve living conditions and create resilient refugee settlements.^9^ Moreover, in conflict countries, there is often a significant reduction in the health system and an increased risk of the spread of infectious diseases like measles, acute respiratory infections and malnutrition. An effective way to address these issues is to enhance access to healthcare by expanding the camp facilities and building medical capacity so that healthcare is provided for all refugees. Also, mass vaccination reduces preventable diseases like measles, particularly among children and high-risk groups. Moreover, effective surveillance of disease outbreaks is crucial in areas affected by drought and flooding, where environmental factors elevate the disease risk.^10^

In conclusion, displacement in Sudan stems from the combined pressures of long-standing conflict, recurrent drought and the severe overcrowding of camps. The urgency of the crisis lies not only in the immediate impact of climate shocks but also in decades of war that have eroded livelihoods, destroyed infrastructure and deepened humanitarian strain. Meeting these challenges calls for stronger health systems, sustainable management of natural resources and climate adaptation measures, alongside renewed efforts at conflict resolution and improved governance. Only through a coordinated response that brings together humanitarian organizations, national authorities and international partners can further deterioration in the lives of displaced communities be averted.

## Data Availability

No new data were generated or analysed to support this research. All the primary sources and articles analysed in this review are cited in the reference section.
